# The microglial P2Y_6_ receptor as a therapeutic target for neurodegenerative diseases

**DOI:** 10.1186/s40035-024-00438-5

**Published:** 2024-09-07

**Authors:** Jacob M. Dundee, Guy C. Brown

**Affiliations:** Department of Biochemistry, University of Cambridge, Cambridge, UK

**Keywords:** Alzheimer’s disease, Parkinson’s disease, Neurodegeneration, Neuroinflammation, P2Y_6_ receptor, Drug development, Microglia

## Abstract

Neurodegenerative diseases are associated with chronic neuroinflammation in the brain, which can result in microglial phagocytosis of live synapses and neurons that may contribute to cognitive deficits and neuronal loss. The microglial P2Y_6_ receptor (P2Y_6_R) is a G-protein coupled receptor, which stimulates microglial phagocytosis when activated by extracellular uridine diphosphate, released by stressed neurons. Knockout or inhibition of P2Y_6_R can prevent neuronal loss in mouse models of Alzheimer’s disease (AD), Parkinson’s disease, epilepsy, neuroinflammation and aging, and prevent cognitive deficits in models of AD, epilepsy and aging. This review summarises the known roles of P2Y_6_R in the physiology and pathology of the brain, and its potential as a therapeutic target to prevent neurodegeneration and other brain pathologies.

## Background

There is increasing evidence that microglia and phagocytosis play important roles in neurodegeneration, for example by microglia phagocytosing synapses and neurons. The microglial P2Y_6_ receptor (P2Y_6_R) regulates microglial phagocytosis, as well as the migration and activation of microglia. The main aim of this article is to review the evidence that inhibition of P2Y_6_R is neuroprotective in models of neurodegeneration and other brain pathologies, and therefore that P2Y_6_R is a promising drug target. We start by outlining the various roles of microglial phagocytosis in brain pathologies. We then introduce P2Y_6_R and its regulation of microglial functions. Subsequently, we review the evidence that inhibition or knockout of P2Y_6_R is protective in models of neuroinflammation, Parkinson’s disease (PD), brain aging, stroke, vascular dementia, epilepsy, Alzheimer’s disease (AD) and non-brain pathologies. We then briefly outline P2Y_6_R pharmacology, and finish by discussing the challenges of targeting P2Y_6_R to treat neurodegenerative diseases.

## Roles of microglial phagocytosis in neurodegeneration

Microglia are macrophages resident in the central nervous system, and they are the main cells mediating immunity, inflammation and phagocytosis in the brain. During development, microglia phagocytose synapses to shape neuronal networks according to experience [1, 2]. Microglia also phagocytose apoptotic neurons and excess live neurons or neuronal precursors during development [3].

The mechanisms of neuronal death in neurodegenerative disease are poorly understood, but there is no evidence of increased apoptosis or necrosis of neurons in these diseases [4], although there is some recent evidence of necroptosis of human neurons in animal models [5]. However, as outlined below, there is accumulating evidence that neuronal loss during neurodegeneration is at least in part mediated by microglial phagocytosis of live neurons, resulting in neuronal cell death by phagocytosis [6]. Cell death by phagocytosis is a very common form of cell death in the body [7] and brain [3, 6, 8]. Extensive synaptic loss also occurs in some neurodegenerative diseases, such as AD, contributing to cognitive deficits, and there is evidence that microglial phagocytosis of synapses contributes to this synaptic loss, at least in animal models of neurodegeneration [1, 9, 10].

Most therapies that have been tried for neurodegenerative diseases target processes early in disease, which has the potential advantage of stopping the disease early, but this has a considerable disadvantage of normally requiring treatment before diagnosis. By contrast, targeting the synaptic and neuronal loss that occur relatively late in these diseases has the potential advantage of stopping disease progression after diagnosis. For example, neuronal loss in AD is a late event correlating with dementia [11], and occurs at least a decade after amyloid plaque deposition and at least 5 years after tau tangles have appeared in the neurons [12]. Delaying neuronal loss for a further 5 years could substantially reduce the progression, prevalence and severity of AD.

AD is the most common neurodegenerative disease, characterised by amyloid β (Aβ) plaques, tau tangles and extensive loss of synapses and neurons. Many of the genes associated with AD risk are mainly expressed by microglia and affect microglial phagocytosis, including *APOE*, *TREM2*, *PLCG2*, *ABI3*, *INPP5D*, *MS4A4A*, *ADAM10*, *ADAM17*, *IL34*, *CTSB*, *CTSH*, *MAF*, *LILRB2*, *ABCA1*, *ABCA7*, *CR1*, *CD33*, *PILRA*, *SIGLEC11*, *CLU*, and *GRN* [13, 14]. APOE can opsonize (i.e., bind to and induce phagocytosis of) synapses, neurons, and Aβ plaques, and then induce microglial phagocytosis via TREM2, PLCγ2 and ABI3, and this pathway of phagocytosis is inhibited by CD33, PILRα, INPP5D/SHIP1, MS4A4A, ADAM10, ADAM17, LILRB2 and SIGLEC11 [13]. Thus, much of the known genetic risk for AD is linked to microglial phagocytosis, but it is unclear whether this is via phagocytosis of soluble Aβ, amyloid plaques, dead cells and debris, or live synapses and neurons. In culture, Aβ or phosphorylated tau can induce microglia to phagocytose live neurons [15–17]. In animal models of AD, inhibition of microglial phagocytosis prevents synaptic or neuronal loss [9, 10], indicating that inhibition of microglial phagocytosis can be beneficial.

Parkinson’s disease (PD) is characterized by motor deficits, Lewy bodies and progressive loss of midbrain dopaminergic neurons. PD risk genes affecting microglial phagocytosis include leucine-rich repeat kinase 2 (*LRRK2*) [18, 19]. Activation of LRRK2 in microglia increases microglial phagocytosis of neuronal processes, which is prevented by *LRRK2* knockdown [18]. The *LRRK2*-G2019S variant associated with PD risk increases microglial phagocytosis of live dopaminergic neurons in culture and in vivo, which is prevented by blocking phagocytosis [20]. Similar results have been found in a *Drosophila* model of PD [20, 21]. *SNCA*, which encodes α-synuclein, is another important PD risk gene. α-Synuclein is the main component of Lewy bodies [22]. α-Synuclein can stimulate microglial phagocytosis [23, 24], and mice expressing A53T α-synuclein have increased microglial expression of Mer and Axl, and knockout of these phagocytic receptors extends survival of the mice [25], suggesting that microglial phagocytosis of neurons contributes to the pathology.

Dopaminergic neurons of the substantia nigra, which are lost in PD, contain high levels of the protein neuromelanin [26]. Extracellular neuromelanin activates microglia and causes neuronal loss, which can be prevented by knockout of the microglial phagocytic receptor CR3 [27], suggesting that microglial phagocytosis of live neurons may cause this neuronal loss. Gut dysfunction occurs early in PD, and may lead to elevated levels of lipopolysaccharide (LPS) endotoxin in the blood of PD patients [28]. In mice, chronic peripheral LPS causes activation of microglia in the substantia nigra, and up-regulation of complement factors and neuronal loss, which can be prevented by complement C3 knockout [29]. Toxins MPTP (1-methyl-4-phenyl-1,2,3,6-tetrahydropyridine) and 6-hydroxydopamine induce microglia to phagocytose dopaminergic neurons in vivo, implicating phagocytosis in dopaminergic degeneration [30–33].

Microglial phagocytosis is also implicated in the pathology of multiple sclerosis, retinal degeneration, stroke, brain viral infections, and brain ageing [34–38]. Thus, there is a need for novel therapies based on inhibiting microglial phagocytosis of live synapses and neurons. P2Y_6_R is one such potential target.

## Introduction to P2Y_6_R

**Fig. 1:**
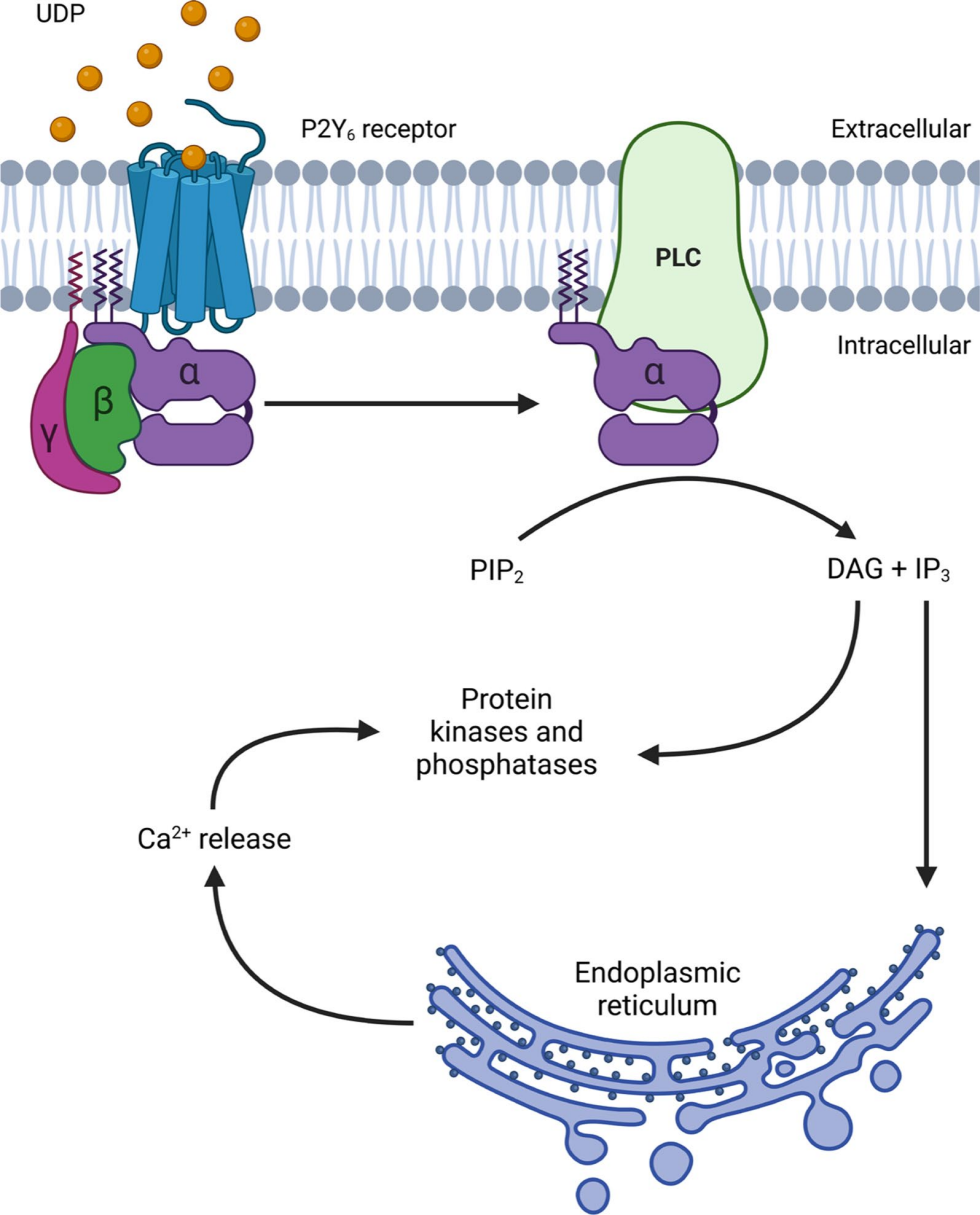
P2Y_6_ receptor (P2Y_6_R) signalling. P2Y_6_R activation by UDP leads to phospholipase C (PLC)-induced conversion of PIP_2_ to diacylglycerol (DAG) and inositol triphosphate (IP_3_), resulting in Ca^2+^ release from the endoplasmic reticulum. Ca^2+^ and DAG activate multiple protein kinases, mediating down-stream signalling. Image created using Biorender

P2Y_6_R is part of the P2Y family of proteins, which has eight members, all being G-protein coupled receptors (GPCRs) for nucleotides [39]. P2Y receptors are either G_q_-coupled receptors (P2Y_1_, P2Y_2_, P2Y_4_, P2Y_6_, and P2Y_11_) or G_i_-coupled receptors (P2Y_12_, P2Y_13_, and P2Y_14_). P2Y_6_R shares 23%–46% of its amino acid sequence with the other P2Y receptors, and is most closely related to P2Y_1_, P2Y_2_, P2Y_4_ and P2Y_11_, which constitute a subfamily of P2Y receptors [39, 40]. P2Y_6_R is found on the plasma membrane. However, similar to other GPCRs, there is evidence that P2Y_6_R can be internalised in a clathrin-dependent manner to regulate activity [41]. The predominant endogenous ligand for P2Y_6_R is extracellular uridine diphosphate (UDP, EC_50_: 50–300 nM), with P2Y_6_R having lower affinity to other nucleotides including uridine triphosphate (UTP, EC_50_: 6 µM), adenosine diphosphate (ADP, EC_50_: 30 µM), and adenosine triphosphate (ATP, EC_50_: 3 mM) [42, 43]. The structure of P2Y_6_R is unsolved, but the structures of P2Y_1_R and P2Y_12_R have been solved [43–45], with the nucleotide-binding site at the extracellular side of transmembrane alpha-helices 1, 3, 6, and 7 [46]. Homology modelling of P2Y_6_R suggests a similar structure with 7 transmembrane alpha helices and a nucleotide-binding site between these, towards the extracellular side [46]. The extracellular loops affect ligand specificity [47]. Upon activation of P2Y_6_R, G_q_ binds GTP and stimulates beta-type phospholipase C to cleave phosphatidylinositol 4,5-bisphosphate (PIP_2_) into diacylglycerol that activates protein kinase Cs and into inositol trisphosphate (IP_3_) that activates IP_3_ receptors in the endoplasmic reticulum, resulting in calcium release into the cytoplasm (Fig. 1).

P2Y_6_R is expressed on multiple cell types throughout the body, particularly in myeloid cells [48]. Within the brain, P2Y_6_R is mainly expressed by microglia [49, 50]. It is also expressed on a very small set of neurons in the hypothalamus that stimulate feeding [51, 52]. Within microglia, activation of P2Y_6_R by extracellular UDP stimulates microglial phagocytosis [53] and may stimulate microglial motility [54].

P2Y_6_R expression by microglia increases in response to acute inflammation and/or excitotoxicity. Kainic acid-induced seizures cause a several-fold increase in P2Y_6_R mRNA in microglia [53, 55]. Li et al. [50] reported that hemorrhagic stroke increased microglial P2Y_6_R protein expression in the mouse brain by 10 folds and P2Y_6_R was exclusively expressed in microglia [50]. Yang et al. [56] found that LPS increased P2Y_6_R mRNA and protein by several folds in cultured BV-2 microglia. They also reported that PD patients had several-fold higher expression of P2Y_6_R in peripheral monocytes, but they did not measure expression in patient microglia. However, in disease-associated microglia or in the context of neurodegenerative diseases, it is unclear whether microglial P2Y_6_R expression changes (http://research-pub.gene.com/BrainMyeloidLandscape/BrainMyeloidLandscape2/).

## UDP and P2Y_6_R in microglia

Koizumi et al. [53] found that P2Y_6_R activation with UDP in primary rat microglia resulted in a 10-fold increase in the phagocytosis of zymosan particles. P2Y_6_R activation induced phagocytosis apparently via actin-reorganisation (filopodia-like protrusions), accumulation of F-actin aggregates (phagosome-like), and the formation of a circular structure (phagocytic cup) observed upon the addition of UDP. This was further supported when P2Y_6_R activation was observed to induce microglial membrane motility and actin aggregation in a protein kinase C-dependent fashion [57, 58]. Wendt et al. [59] reported that the UDP-induced phagocytosis was reduced in plaque-associated microglia, but there was a normal response to UDP in non-plaque-associated microglia in brain slices from amyloid mice.

Langfelder et al. [54] reported that, in a microglial cell line, P2Y_6_R activation induced microglial migration/motility, measured by the scratch assay. As microglial motility and migration are necessary for microglial phagocytosis, this increase in motility may contribute to the P2Y_6_R stimulation of phagocytosis. Additionally, Kim et al. [60] reported that UDP induced expression and release of two chemokines (CCL2/MCP-1 and CCL3/MIP-1a) from primary microglia (and astrocytes) via activation of P2Y_6_R. These chemokines can recruit monocytes into the injured brain [60], but also recruit microglia [61]. Timmerman et al. [62] found that P2Y_6_R signalling increased the pro-inflammatory response of microglia to Toll-like receptor (TLR) activation, and inhibition of P2Y_6_R by MRS2578 reduced this. Yang et al. [56] found the same and attributed the enhanced LPS response to activation of ERK1/2 by UDP/P2Y_6_R. Umpierre et al. [55] reported that P2Y_6_R knockout in mice reduced the induction of NF-κB-dependent inflammatory genes (as well as phagocytosis) in microglia in response to seizures. Thus, inhibition of P2Y_6_R potentially reduces microglia recruitment, activation and phagocytosis.

What causes UDP release in the brain? Koizumi et al. [53] found that treatment of primary neurons in vitro with kainic acid (to activate excitatory glutamate receptors) induces the release of UTP, which is broken down to UDP by extracellular ectonucleotidases. Kainic acid injected intraperitoneally into rats also caused a 10-fold increase in extracellular UTP level in the CA3 region of the hippocampus. Thus, excitatory stress appears to induce neuronal release of UTP, which is broken down to UDP, inducing P2Y_6_R-dependent phagocytosis. This is supported by Umpierre et al. [55] reporting that kainic acid induces rapid, transient and localised elevations of extracellular UDP in mouse brain, in addition to sustained increases in UDP in response to kainic acid, resulting in microglial phagocytosis of neurons and cognitive deficits prevented by P2Y_6_R knockout. Yang et al. [56] found that LPS increased extracellular UDP levels outside cultured BV-2 microglia by unknown means, so it may be that inflammation also increases extracellular UDP.

**Fig. 2:**
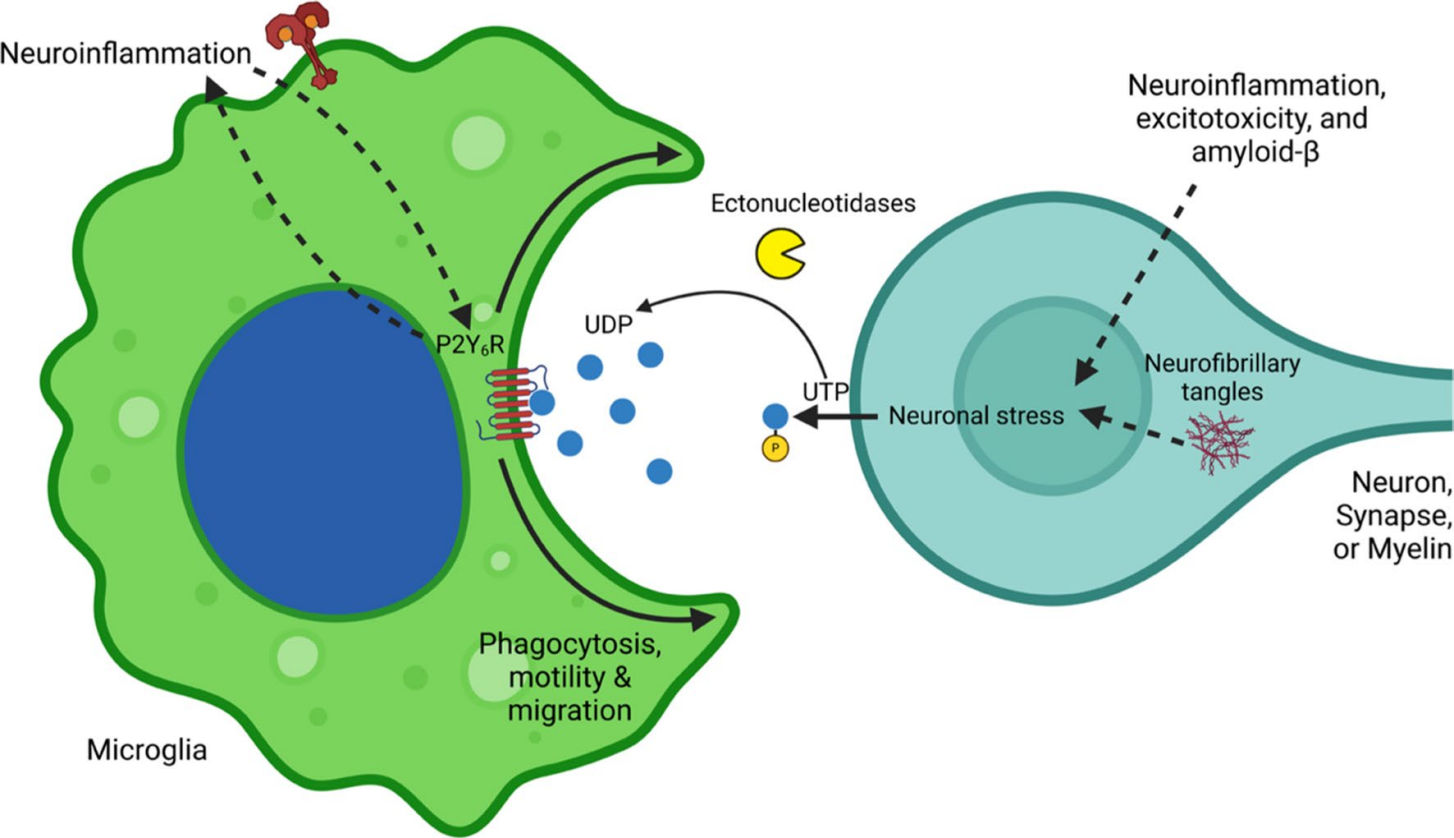
Mechanisms of P2Y_6_ receptor (P2Y_6_R)-mediated neurodegeneration. Stressed neurons (and glia) can release UTP, which is degraded by ectonucleotidases to UDP, which activates P2Y_6_R on microglia to induce microglial phagocytosis of neurons or synapses. Image created using Biorender

UTP can be released (together with ATP) from a wide variety of cells (including astrocytes) when activated by, for example, mechanical stimulation [63]. The mechanism of release is unclear, but in the case of ATP release it is generally by vesicular exocytosis, volume-activated anion channels, or connexin or pannexin hemichannels [64]. Both UTP and ATP can be transported into vesicles, including synaptic vesicles, by the vesicular nucleotide transporter [64], potentially enabling synaptic release of UTP by vesicular exocytosis, but this has not been tested directly. Extracellular UTP can be converted to UDP by a variety of extracellular nucleotidases, of which the main one expressed on the surface of microglia is CD39, which can both convert UTP to UDP and convert UDP to UMP [65]. Other nucleotidases are expressed on neurons, astrocytes and microglia, some of which generate the P2Y_6_R ligand UDP from UTP, and others degrade UDP [65]. Overall, the evidence suggests that stressed neurons (and other cells) release UTP that degrades to UDP, which activates P2Y_6_R to induce microglial phagocytosis of synapses or neurons (Fig. 2). However, UDP release is still poorly understood. UDP may come from neurons, astrocytes and microglia, when stressed, activated or inflamed, and as each of these conditions changes during neurodegeneration [1, 11, 66], it is difficult to predict extracellular UDP levels. Hence, it would be informative to measure extracellular UDP levels during neurodegeneration.

## P2Y_6_R in inflammation-induced neurodegeneration and models of PD

According to the above evidence, UTP can be released from stressed neurons, and then converted to UDP, which activates microglial phagocytosis—but can this result in microglial phagocytosis of stressed neurons? Neher et al. [67] reported that UDP increased microglial phagocytosis, and addition of UDP to primary co-cultures of neurons, astrocytes and microglia induced neuronal loss, which can be prevented by inhibition of P2Y_6_R, microglial depletion or inhibition of phagocytosis, implying that UDP induces microglia to phagocytose live neurons. Knockout of P2Y_6_R also prevents the UDP-induced neuronal loss [68]. Emmrich et al. [69] found that blocking P2Y_6_R prevented rotenone-induced neuronal loss in these co-cultures (an in vitro model of PD) by reducing microglial phagocytosis. And Neniskyte et al. [70] found that TNF-α-induced neuronal loss in these co-cultures could be prevented by blocking P2Y_6_R.

LPS, which activates microglia via TLR4, can induce increased P2Y_6_R expression by cultured microglia, as well as UDP release [56]. In glial-neuronal co-cultures, LPS induces microglia to phagocytose neurons [71], resulting in neuronal loss in these cultures, which can be prevented by the addition of either MRS2578, a specific P2Y_6_R inhibitor, or apyrase that degrades nucleotides such as UDP, suggesting that inflammation-induced neuronal loss is mediated by P2Y_6_R [67]. Injection of LPS into the striatum of rats was found to induce microglial engulfment of neurons and subsequent neuronal loss, both of which were inhibited by co-injection of MRS2578 to block P2Y_6_R [67]. Hornik, Vilalta and Brown [72] later found that activation of P2Y_6_R increased BV-2 phagocytosis, while inhibition of P2Y_6_R reduced phagocytosis of PC12 neuronal-like cells by LPS-treated BV-2 microglia. Milde et al. [73] found that glial-neuronal cultures from P2Y_6_R knockout mice had reduced LPS-induced neuronal loss compared to co-cultures from wild-type mice.

Brain inflammation, including microglial activation, is present in most brain pathologies, including PD, and LPS is commonly used to model this microglial activation and neuroinflammation in experimental systems. However, there is recent evidence that LPS may be causally involved in neurodegenerative diseases, particularly PD, as blood LPS levels are elevated in PD patients, probably due to increased gut permeability [28, 74, 75]. LPS increases P2Y_6_R expression in microglia, and PD patients have several-fold higher expression of P2Y_6_R in peripheral monocytes [56]. Injection of LPS intraperitoneally into mice daily for 4 days resulted in a loss of dopaminergic neurons specifically in the substantia nigra of wild-type mice (i.e., the neuronal population specifically lost in PD), but this LPS-induced neuronal loss was absent in P2Y_6_R-knockout mice [73]. This suggests that blocking P2Y_6_R might prevent the inflammatory neuronal loss in PD. Supporting this, Oliveira-Giacomelli et al. [76] found that the P2Y_6_R antagonist MRS2578 prevented neuronal loss in substantia nigra of mice in the 6-hyrdoxydopamine model of PD.

## P2Y_6_R in aging-induced loss of synapses and memory

During aging, there is chronic, low-level neuroinflammation in the brain and inflammatory activation of microglia [77]. Brain aging also leads to synaptic loss in both mice and humans, and complement-mediated microglial phagocytosis of synapses is implicated in this aging-induced synaptic loss [35]. P2Y_6_R may mediate in part microglial phagocytosis of synapses, as indicated by the findings of Dundee et al. [78] that inactivation of P2Y_6_R decreased microglial phagocytosis of isolated synapses (synaptosomes) and synaptic loss in neuronal-glial co-cultures. In vivo, it was found that microglial phagocytosis of synapses was increased in the brains of aged wild-type mice, but this increase was absent in P2Y_6_R-knockout mice. P2Y_6_R-knockout mice were also protected from aging-associated loss of synapses and memory [78]. This work indicates that inhibiting P2Y_6_R can prevent memory loss with age in mice, probably by preventing microglial phagocytosis of synapses, and that long-term inhibition of P2Y_6_R is not detrimental to the brain, at least in mice. However, Dundee et al. [79] recently reported that young P2Y_6_R-knockout mice had reduced microglial phagocytosis of synapses and impairment of memory, indicating that P2Y_6_R may contribute to microglial phagocytosis of synapses during development. It is unclear whether P2Y_6_R might affect memory in adult mice, but P2Y_6_R knockout mice retain memory better with age [78]. As P2Y_6_R knockout or inhibition is also beneficial for a wide variety of age-related diseases in mice, P2Y_6_R antagonists might be beneficial in human aging [80].

## P2Y_6_R in models of stroke, vascular dementia and epilepsy

Transient or chronic ischemia can induce neuronal loss by multiple mechanisms during stroke or vascular dementia [4]. The brain areas suffering the strongest ischemia usually experience neuronal death quickly by necrosis and form an infarct that is cleared over time through microglial phagocytosis of dead cells and debris [37, 81]. However, brain areas suffering less ischemia (the penumbra) or areas connected by axons to the infarct may have delayed neuronal death, and there is evidence that this is in part mediated by microglial phagocytosis of stressed-but-viable neurons [37, 81].

Wen et al. [82] found that P2Y_6_R inhibition by MRS2578 worsened brain damage and function after middle cerebral artery occlusion in mice (a model of severe stroke), apparently due to reduced microglial phagocytosis of dead cells and debris. A similar conclusion was reached by Xu et al. [83], who irradiated mice with β radiation (a model of brain damage) and subsequently exposed them to a P2Y_6_R inhibitor, which increased the density of apoptotic neurons and myelin damage. In contrast, Li et al. [50] found that P2Y_6_R inhibition by MRS2578 reduced brain damage and improved neurological outcome in a mouse model of hemorrhagic stroke, but they attributed the reduced damage to reduced microglial pyroptosis and inflammation. Li et al. [50] also reported that hemorrhagic stroke increased P2Y_6_R protein level by 10 folds and P2Y_6_R was exclusively expressed in microglia. Milde and Brown [84] found that P2Y_6_R-knockout mice had reduced microglial phagocytosis of neurons and no significant neuronal loss in peri-infarct brain areas after mild, transient stroke. Thus, whether P2Y_6_R inhibition is beneficial or detrimental may depend on the degree and the type of brain ischemia/damage: P2Y_6_R-dependent microglial phagocytosis may be beneficial in severe brain damage by removing debris and remodeling of what remains, whereas it may be detrimental with less severe ischemia/damage where microglia may phagocytose stressed-but-viable neurons. However, the timing of inhibition may also be critical, and more research is required to test when and in what conditions P2Y_6_R inhibition is beneficial.

In models of epilepsy, Umpierre et al. [55] found that kainic acid-induced seizures or excitotoxity caused rapid UDP release in the mouse brain, stimulating P2Y_6_R-dependent calcium transients in microglia. This was followed by a several-fold increase in P2Y_6_R mRNA in microglia and sustained UDP release, resulting in P2Y_6_R-dependent: inflammatory activation of microglia, monocyte recruitment into brain, increased microglial lysosomes and microglial engulfment of whole neurons [55]. The resulting neuronal loss and cognitive deficits were prevented by P2Y_6_R knockout, suggesting that epilepsy- or excitotoxicity-induced brain damage may be reduced by inhibition of P2Y_6_R [55].

## P2Y_6_R in models of AD

AD brains are characterised by amyloid plaques (extracellular aggregates of Aβ) and tau tangles (intraneuronal aggregates of hyperphosphorylated tau), together with neuroinflammation and extensive loss of synapses and neurons. Addition of Aβ to co-cultures of glia and neurons resulted in a loss of synapses and neurons mediated by microglial phagocytosis [15, 71]. Inhibition or knockout of P2Y_6_R reduced this neuronal loss, consistent with Aβ induction of microglial phagocytosis of stressed-but-viable neurons [67, 68]. Addition of Aβ to neuronal-like PC12 cells induced UDP release without killing the cells [68], consistent with Aβ-stressed neurons releasing UTP/UDP. Addition of tau to co-cultures of glia and neurons also resulted in neuronal loss, mediated by microglial phagocytosis [17] and prevented by inhibition of P2Y_6_R [68].

Puigdellívol et al. [68] found that stereotactic injection of aggregated Aβ into the brain induced microglial phagocytosis of neurons, as indicated by uptake of neuronal nuclear material into the microglia, but this uptake was greatly reduced in P2Y_6_R-knockout mice. This reduced microglial phagocytosis of neurons prevented Aβ-induced neuronal and memory loss in P2Y_6_R-knockout mice. Similarly, transgenic mice expressing P301S *MAPT* and thus chronic tauopathy, had neuronal and memory loss that was prevented by crossing with P2Y_6_R knockout mice. However, the neuronal loss in this model was modest and this loss was only partially reduced in P2Y_6_R-knockout mice, whereas the memory loss was completely prevented. Thus, P2Y_6_R knockout may protect against tauopathy-induced neurodegeneration by more than one means. Overall, these studies indicate that P2Y_6_R inhibition may be useful in preventing neurodegeneration.

Others have suggested that the activation of P2Y_6_R could be important for microglial clearance of amyloid plaques in AD [85] and therefore the use of agonists to P2Y_6_R could ameliorate symptoms through removal of these plaques. GC-021109 is one such agonist that has been tested by the company Gliacure in phase 1 trials (NCT02254369 and NCT02386306), although there is no peer-reviewed data supporting P2Y_6_R-dependent microglial phagocytosis of amyloid plaques [85]. If P2Y_6_R agonists do induce microglial phagocytosis of amyloid plaques, there is a danger that they may also induce microglial phagocytosis of viable synapses and neurons, but this has not been tested.

Could P2Y_6_R antagonists be detrimental by inhibiting microglial phagocytosis of amyloid plaques and neuronal debris? This partly depends on whether there is sufficient extracellular UDP present to induce this phagocytosis. Amyloid plaques and neuronal debris do not release UTP/UDP, whereas stressed and dying neurons can, so P2Y_6_R antagonists should not inhibit microglial phagocytosis of amyloid plaques and neuronal debris. Furthermore, the empirical findings are that P2Y_6_R knockout is beneficial in amyloid and tau models of neurodegeneration [68].

P2Y_6_R antagonists might also be detrimental by inhibiting immunity in brain and body; however, there is no current evidence that P2Y_6_R knockout mice are more susceptible to infections. P2Y_6_R antagonists might alternatively be detrimental by inhibiting microglial phagocytosis of debris in the brain, but there is no evidence for this, probably because debris does not release UDP [68]. However, if P2Y_6_R antagonists rescue neurons with tau aggregates this might be detrimental in the longer term by (1) allowing dysfunctional neurons to survive that are detrimental to neuronal networks, and (2) allowing tau aggregates to be released and spread through the brain. On the other hand, tau spreading may in part be mediated by microglial phagocytosis of live neurons with tau aggregates [86], so blocking this with P2Y_6_R antagonists might slow tau spreading. And again, the empirical finding is that P2Y_6_R knockout is beneficial to both neuropathology and cognition in amyloid and tau models of neurodegeneration [68], so P2Y_6_R inhibition appears to be of net benefit in these models.

## P2Y_6_R as a therapeutic target for non-brain pathologies

P2Y_6_R is expressed on multiple cell types, throughout the body, particularly on myeloid cells [48]. Therefore, it has potential roles in pathologies outside the brain. Knockout or inhibition of P2Y_6_R can reduce a variety of non-brain pathologies in mouse models, including hypertension [87], atherosclerosis [88, 89], heart failure [90], obesity [51], diabetes [52], fatty liver disease [91], inflammatory bowel disease [92, 93], neuropathic pain [94], asthma [95], cancer [96, 97], pulmonary fibrosis [98], and pulmonary edema [93]. P2Y_6_R inhibition has been suggested to be beneficial against hypertension and cardiovascular diseases via inhibition of angiotensin signaling [99]. Inhibition of P2Y_6_R is also thought to be beneficial against many of these pathologies by reducing inflammation, as P2Y_6_R inhibition reduces the release of chemokines and cytokines from innate immune cells [100, 101], and reduces migration of innate immune cells to the site of damage [102]. In obesity, a small subset of neurons in the hypothalamus express P2Y_6_R, which increases feeding behaviour in response to local UDP, and P2Y_6_R knockout mice are resistant to excessive feeding, suggesting that UDP and P2Y_6_R mediate excessive feeding in obesity [51, 52]. P2Y_6_R knockout mice are also protected against diet-induced obesity, having improved glucose tolerance and insulin sensitivity with reduced systemic inflammation, suggesting that P2Y_6_R antagonists might be used for treatment of obesity and type 2 diabetes [52, 103]. However, P2Y_6_R knockout appears detrimental in some mouse models of inflammatory bowel disease [104] and glaucoma [105]. Overall, inhibition or knockout of P2Y_6_R appears protective in mouse models of a remarkably-wide range of pathologies, but may be counter-indicated in glaucoma.

As P2Y_6_R inhibition is beneficial in a wide range of non-brain pathologies, it is worth considering whether these non-brain effects may contribute to protection against brain pathologies. Hypertension and atherosclerosis contribute to stroke and vascular dementia; therefore, P2Y_6_R inhibition might be beneficial by reducing hypertension and atherosclerosis. Obesity and type 2 diabetes predispose to dementia, and therefore P2Y_6_R inhibition might be beneficial by reducing these. Inflammatory bowel disease may predispose to PD, so P2Y_6_R inhibition might affect PD risk via affecting inflammatory bowel disease; however, opposite effects of P2Y_6_R inhibition on models of inflammatory bowel disease have been reported [92, 104].

## Current P2Y_6_R inhibitors

The most commonly used inhibitor of P2Y_6_R is MRS2578, which covalently binds to intracellular residues on P2Y_6_R, resulting in internalisation of P2Y_6_R [106, 107], with an apparent IC_50_ of 37 nM for human P2Y_6_R [108]. However, MRS2578 is unlikely to be a usable therapeutic due to its solubility, stability, toxicity and mode of inhibition [108, 109].

Other antagonists include a number of nitro-benzopyran compounds, including TIM-38, MRS4774, and MRS4853 with IC_50_ values of 4 µM, 0.6 µM, and 0.5 µM, respectively [110–112]. Recently, a class of derivatives of 2-(1-(tert-butyl)-5-(furan-2-yl)-1 H-pyrazol-3-yl)-1 H-benzo[d]imidazole have been described, including compound 50, with an IC_50_ of 6 nM and specificity to P2Y_6_R, which protected mice from ulcerative colitis and LPS-induced lung injury [93]. However, it is unclear whether these compounds can cross the blood-brain barrier or have toxicity. Developing useable P2Y_6_R inhibitors that can cross the blood-brain barrier to inhibit microglial phagocytosis without toxicity is essential to test the therapeutic potential of P2Y_6_R in neurodegeneration.

## Conclusion

There is growing evidence that P2Y_6_R and microglial phagocytosis mediate neurodegeneration, so inhibiting P2Y_6_R can be beneficial. However, we are still lacking practical inhibitors of microglial P2Y_6_R, which could be used to further validate the target prior to clinical trials.

## Data Availability

Not applicable.

## Abbreviations

AD: Alzheimer’s disease
ATP: Adenine triphosphate
IP_3_: Inositol triphosphate
LPS: Lipopolysaccharide
LRRK2: Leucine-rich repeat kinase 2
P2Y_6_R: P2Y_6_ receptor
PD: Parkinson’s disease
PIP_2_: Phosphatidylinositol 4,5-bisphosphate
UDP: Uridine diphosphate
UTP: Uridine triphosphate
